# Editorial: Herpesvirus: transmission, pathogenesis, host-pathogen interaction, and treatment

**DOI:** 10.3389/fmicb.2025.1637344

**Published:** 2025-06-17

**Authors:** Tejabhiram Yadavalli, Sita Awasthi, Jinlin Li

**Affiliations:** ^1^Department of Ophthalmology and Visual Sciences, University of Illinois Chicago, Chicago, IL, United States; ^2^Infectious Disease Division, Department of Medicine, Perelman School of Medicine, University of Pennsylvania, Philadelphia, PA, United States; ^3^Department of Medical Biochemistry and Microbiology, Zoonosis Science Center, Uppsala University, Uppsala, Sweden

**Keywords:** EBV, HSV-1, BHV 1, HSV-2, CMV, HHV-7, pulmonary infection

Herpesviruses are a large group of viruses including the members that can infect humans, bovines, birds, and a wide range of other hosts. These viruses have large double-stranded DNA genomes and express a large of envelope, tegument, and capsid proteins. At least five of the nine known human herpesviruses are particularly widespread, including Epstein-Barr virus (EBV), Herpes simplex virus 1 and 2 (HSV-1 and HSV-2), Varicella zoster virus (VZV), and Human cytomegalovirus (HCMV) (Grinde, 2013). In addition to human herpesviruses, one of the most important viruses linked to cattle diseases is Bovine herpesvirus 1 (BHV1) (Nandi et al., 2009). This Research Topic explores the transmission, pathogenesis, host-pathogen interactions, and treatment of herpesviruses.

Human herpesvirus reactivation remains an extremely interesting and complex topic of research. The EBV trans-activator, ZEBRA (also known as Zta, Z, EB1, encoded by EBV gene BZLF1) was discovered 40 years ago (Countryman and Miller, 1985), and known to play an important role in regulating EBV reactivation. In this Research Topic, Wang et al. comprehensively summarized the key factors that regulate the reactivation of EBV at the different stages of the lytic cycle including the immediate-early (IE) genes expression, the DNA replication, and progeny virus production. They discussed the regulation of the viral immediate-early proteins ZEBRA and Rta at the transcriptional levels by various activators, repressors, and epigenetic modifications. Additionally, they pointed out the essential roles of the host partners and modifications in remodeling ZEBRA and Rta activities. The authors summarized the regulation of the EBV lytic cycle after the initiation by different EBV-encoded proteins, viral miRNAs, and host factors, and discussed the emerging tools and technologies, such as structural prediction tools (Alphafold3), literature data mining tools [Natural Language Processing (NLP)] and genome editing tools (CRISPRx, derived from CRISPR systems) for advancing our understanding of mechanisms of latency and reactivation.

The research article from Ripa et al. investigated the interaction between HSV-1 and autophagy in glial cells and found that HSV-1 inhibited the formation of autophagosomes. Knocking out the ATG5 gene in the HOG and U-87 MG cell lines using the CRISPR/Cas9 led to the suppression of HSV-1 transcription and replication. No significant differences in viral production were observed when knocking out the MAP1LC3B gene. Based on these results, Ripa et al. proposed that HSV-1 hijacks non-canonical functions of certain components of the autophagic machinery, such as ATG-5, to facilitate its replication by inhibiting the complete autophagy in glial cells. Shi et al. established an HSV-2 infection model using HaCaT cells and evaluated the potential role of ACV in treating HSV-2 infection by enhancing the host antiviral immune response through regulation of the TLR9 signaling pathway.

Another interesting topic related to herpesviruses and pulmonary infections was investigated by Liu et al.. By employing metagenomic next-generation sequencing (mNGS) of 100 respiratory samples from patients with pulmonary infection, they detected a total of 43 bacterial species, 12 fungal species, and 5 viral species. EBV, *Candida albicans*, and *Haemophilus parainfluenzae* are the most frequently detected viruses, fungi, and bacteria, respectively. It is worth noting that herpesviruses were the only DNA viruses detected. The average hospitalization duration was significantly longer for patients who tested positive for herpesvirus compared to those who tested negative. The patients who tested positive for viral infection were more likely to have co-infections with other pathogens, with *Pneumocystis jirovecii* and *Aspergillus fumigatus* being the most commonly identified.

Significant disparities in microbial community composition were observed between the virus-positive and virus-negative groups. Through the analysis of the correlation between herpesvirus and high abundance species, a distinct positive correlation was observed between *Haemophilus parainfluenzae* and three herpesviruses: EBV, CMV, and HHV-7. This study pointed out the important association of active herpesvirus and pulmonary infections.

In addition to the human herpesviruses, in this Research Topic, Yan et al. studied the potential role of *Serratia marcescens* on BHV1 infection. *Serratia marcescens* is a Gram-negative bacterium frequently found in a wide range of environments and commonly co-infected with BHV1. Yan et al. assessed the effects of recombinant serralysin-like protease D (rSPD) on BHV1 infection. Serralysin-like protease D is an extracellular enzyme secreted by *Serratia marcescens*. They found that rSPD significantly promoted BHV1 production in Madin-Darby bovine kidney (MDBK) cells. Furthermore, the transcriptomic analysis showed that rSPD curbs innate immune responses, evidenced by the downregulation of innate immunity-associated genes, such as *ISG15, OAS2, IFIT1, IFIT2, IFIT3, MX1, RSAD2, MX2, SAA3, DDX58, IFI44*, and *IRF1*. In addition, rSPD was found to upregulate the genes associated with inflammatory response, including *IL-6, IL-8, CCL2, CX3CL1, CCL3*, and *CXCL3 which may increase cell damage*. Based on these results, Yan et al. propose that rSPD may enhance BHV-1 replication by suppressing the expression of antiviral genes and promoting viral spread through upregulated inflammatory responses.

In summary, the articles in this Research Topic will enhance our understanding of herpesviruses and associated diseases.
